# A multi-trait GWAS-based genetic association network controlling soybean architecture and seed traits

**DOI:** 10.3389/fpls.2023.1302359

**Published:** 2024-01-08

**Authors:** Mengrou Niu, Kewei Tian, Qiang Chen, Chunyan Yang, Mengchen Zhang, Shiyong Sun, Xuelu Wang

**Affiliations:** ^1^ Center of Integrative Biology, College of Life Science and Technology, Huazhong Agricultural University, Wuhan, Hubei, China; ^2^ Sanya Institute of Henan University, Henan University, Sanya, Hainan, China; ^3^ State Key Laboratory of Crop Stress Adaptation and Improvement, School of Life Science, Henan University, Zhengzhou, China; ^4^ The Academy for Advanced Interdisciplinary Studies, Henan University, Zhengzhou, China; ^5^ Institute of Cereal and Oil Crops, Hebei Academy of Agriculture and Forestry Sciences/Hebei Laboratory of Crop Genetics and Breeding, Shijiazhuang, Hebei, China

**Keywords:** soybean, agronomic traits, architecture, GWAS, branch angle

## Abstract

Ideal plant architecture is essential for enhancing crop yields. Ideal soybean (*Glycine max*) architecture encompasses an appropriate plant height, increased node number, moderate seed weight, and compact architecture with smaller branch angles for growth under high-density planting. However, the functional genes regulating plant architecture are far not fully understood in soybean. In this study, we investigated the genetic basis of 12 agronomic traits in a panel of 496 soybean accessions with a wide geographical distribution in China. Analysis of phenotypic changes in 148 historical elite soybean varieties indicated that seed-related traits have mainly been improved over the past 60 years, with targeting plant architecture traits having the potential to further improve yields in future soybean breeding programs. In a genome-wide association study (GWAS) of 12 traits, we detected 169 significantly associated loci, of which 61 overlapped with previously reported loci and 108 new loci. By integrating the GWAS loci for different traits, we constructed a genetic association network and identified 90 loci that were associated with a single trait and 79 loci with pleiotropic effects. Of these 79 loci, 7 hub-nodes were strongly linked to at least three related agronomic traits. qHub_5, containing the previously characterized *Determinate 1* (*Dt1*) locus, was associated not only with plant height and node number (as determined previously), but also with internode length and pod range. Furthermore, we identified qHub_7, which controls three branch angle-related traits; the candidate genes in this locus may be beneficial for breeding soybean with compact architecture. These findings provide insights into the genetic relationships among 12 important agronomic traits in soybean. In addition, these studies uncover valuable loci for further functional gene studies and will facilitate molecular design breeding of soybean architecture.

## Introduction

Soybean is one of the most important oilseed crops worldwide (https://soystas.com). However, the increase in soybean production cannot keep up with the rising demand (Ray et al., 2013). Therefore, increasing yields remains the major aim of soybean breeding. Manipulating plant architecture is an essential strategy for increasing crop yields (Hedden, 2003; Tian et al., 2019; Zhang et al., 2022). Significant progress has been made in improving plant architecture in major cereal crops by targeting specific genes, including the major genes responsible for plant height and leaf angle, such as the “Green Revolution” genes *Reduced height* (*Rht-B1b*) in wheat (*Triticum aestivum*), *semi-dwarf 1*(*sd1*) in rice (*Oryza sativa*) and the “upright plant architecture” genes *UPA1* and *UPA2* in maize (*Zea mays*) (Peng et al., 1999; Sasaki et al., 2002; Tian et al., 2019). Soybean is a typical pod crop, with pods growing at each node all over the plant. Thus, soybean architecture is different from that of graminaceous crops. Previous studies have proposed that ideal soybean architecture encompasses an appropriate plant height, short internodes, high podding rates, a high ratio of four-seeded pods, small petiole angles, short petiole length, increased internode production, and moderate seed size (Liu et al., 2020). In addition to these traits associated with ideal architecture in soybean, erect branches have long attracted the attention of breeders due to the substantial contribution of this trait to soybean yield under dense planting (Li et al., 2019; Clark et al., 2021). However, only a few genes involved in regulating these traits have been characterized (Liu et al., 2010; Ping et al., 2014; Gao et al., 2017).

The ideal goal of crop improvement is to aggregate multiple desirable agronomic traits into a single variety, which requires the simultaneous selection and improvement of numerous traits during breeding. However, close phenotypic correlations among agronomic traits may lead to the co-selection of undesirable traits when screening for a favorable trait, especially when the target traits are negatively correlated (Rharrabti et al., 2001; Rotundo et al., 2009; Chen and Lubberstedt, 2010). For instance, the rice *small grain2* (*sg2*) mutant exhibits both decreased plant height and undesirably small grains (Huang et al., 2022). The rice *pi21* mutant shows durable disease resistance, but this locus is closely linked to a gene conferring poor flavor (Fukuoka et al., 2009). Therefore, pleiotropy of a locus or close linkage between different loci imposes strong barriers to crop improvement. Thus, exploring the genetic relationships among different agronomic traits will increase our understanding of the genetic and molecular basis of these traits, and such information is essential for designing soybean with ideal architecture.

In this study, we used a panel of 496 diverse soybean cultivars, as previously reported (Zhang et al., 2021), cultivated them in two geographical locations for three years, and phenotyped them for 12 important agronomic traits. We identified 169 significantly associated genetic loci with 108 newly identified loci and 61 loci co-located with previously identified quantitative trait loci (QTLs), using GWAS. We also conducted genetic association network analysis of these 12 agronomic traits and identified 90 independent nodes linked to one trait, 26 nodes linked to two traits and 7 hub-nodes linked to three or more agronomic traits. In addition, we predicted two candidate molecular modules (hub-nodes) associated with plant height-related traits and branch angle-related traits. Our findings provide valuable genetic loci and uncover genetic relationships between soybean architecture and seed traits.

## Materials and methods

### Plant materials, phenotypic data collection and quality control

A natural population of 496 soybean accessions was used in this study, consisting of 280 landraces and 216 improved cultivars with a wide geographical distribution in China, and identified 9,357,842 single-nucleotide polymorphisms (SNPs). Principal component analysis of above SNPs showed a continuous distribution without distinct clusters. The linkage disequilibrium (LD) decreased to half of its maximum value at 455 kb for all samples (Zhang et al., 2021). The 148 historical soybean varieties were selected from 216 improved soybean lines, and origin, soybean-growing region and releasing years used in this study were collected on the basis of public registration information or personal communication with soybean breeders (detailed information was listed in **Supplementary Table 1**).

This experiment was carried out simultaneously at the Dishang experimental station of the Institute of Cereal and Oil Crops, Hebei Academy of Agricultural and Forestry Sciences (Shijiazhuang) and the crop experimental base of Huazhong Agricultural University (Wuhan) from 2016 to 2018 (detailed information was listed in **Supplementary Table 2**). Normal full seeds were selected for sowing in both environments, with three rows in each plot, each row being 1 meter long, with 0.4 meters between rows, and 0.1 meter between plants. Three repeats were set in each experimental environment using a randomized block design. At three weeks after sowing, the overall planting density was manually thinned to ~ 250,000 plants per hectare.

The same measurement methods and standards (as listed in **Supplementary Table 3**) were used to investigate agronomic traits under all experimental conditions. Twelve agronomic traits were investigated, comprising nine plant architecture-related traits and three seed-related traits. The plant architecture traits were measured after plant maturity, and seed traits were measured after harvest. Seed length, seed width, and hundred-seed weight were measured using mixed samples harvested in each plot. Ten healthy seeds were randomly selected and measured with a vernier caliper. Hundred-seed weight was measured by weighing 100 healthy seeds harvested in each plot using a balance. The 1^st^ branch angle, 2^nd^ branch angle, 3^rd^ branch angle, seed length, seed width, and hundred-seed weight were measured at both Shijiazhuang and Wuhan experimental stations only in 2016. Plant height, node number, lowest pod height, and branch number were measured in 2016, 2017, and 2018 at both experimental stations. Pod range was calculated by dividing plant height minus lowest pod height by plant height, and internode length was calculated by dividing plant height by node number. The R package “lme4” to was used to calculate the best linear unbiased estimator (BLUE) values and broad-sense heritability for phenotypic data from multiple environments for GWAS.

We estimated the variance of genotype, environment, and the interactions between genotype and environment for plant height (PH), node number (NN), internode length (IL), pod range (PR), lowest pod height (LPH), the 1^st^ branch angle (1-BA), the 2^nd^ branch angle (2-BA), the 3^rd^ branch angle (3-BA), and branch number (BN) by analysis of variance (ANOVA). We only estimated the variance of genotype and environment for hundred-seed weight (HSW), seed width (SW), and seed length (SL) for these traits using mixed samples grown in Shijiazhuang in 2016.

### Genome-wide association study

GWAS was performed based on phenotypic data for agronomic traits in plants from multiple environments (E1 to E6) and BLUE data for each trait, using the genotypic dataset as previously reported (Zhang et al., 2021). A multi-locus mixed-model method (MLMM) model in the R package “GAPIT3” was used as the GWAS model to detect minor-effect genomic regions, the kinship matrix, and the first ten principal components as co-variants (Segura et al., 2012; Wang and Zhang, 2021).

Significantly associated variants were binned together into peaks using a clumping method using the PLINK (Version 1.90) command plink –clump –clump-p1 1E-5 –clump-p2 1E-3 –clump-kb 455 for clump analysis as previously reported (Crowell et al., 2016). A clump was regarded as a singleton if fewer than five single nucleotide polymorphisms (SNPs) with –log10(p)<2.5 × 10^–4^ were present within the clump interval, and the genome coordinate interval of each clump was defined as a GWAS QTL.

All accessible GWAS QTL and mapping QTL information were collected from SoyBase (https://www.soybase.org/). All QTL pairs with overlapping genome intervals were considered to be co-located QTL pairs.

### Construction of the genetic association network

To evaluate the direct genetic correlations of QTLs, all QTL pairs located on the same chromosome in the GWAS results with all phenotypic BLUE data were identified; the Inter-LD between QTLs was calculated using the following formula as previously reported (Fang et al., 2017):


$$Inter-LD=1/2\times (\frac{aveLD(QTL1,QTL2)}{P\max LD(QTL1)}+\frac{aveLD(QTL1,QTL2)}{P\max LD(QTL2)})$$

,where aveLD (QTL1, QTL2) is the mean value of linkage disequilibrium (LD) between all variants in the interval of QTL1 and all variants in the interval of QTL2, and PmaxLD (QTL1) is the maximum value of the mean value of LD between any two variants in QTL1. PmaxLD (QTL2) is the maximum value of the average LD values of any two variants within QTL2. LD is the r^2^ value between the specific SNPs calculated using PLINK. All QTL pairs with Inter-LD≥0.4 were screened. Cytoscape (Version 3.7.2) was used to construct the genetic association network in which agronomic traits and QTLs as nodes and QTL LD as edges are represented by dashed lines. Node diameter represents the minimum *p*-value of each variant in the QTL.

### Candidate gene analysis

All genes in the overlapping region of GWAS results for branch angle related traits were extracted from the Williams 82 reference genome (https://phytozome-next.jgi.doe.gov/info/Gmax_Wm82_a2_v1). R function modified from R package IntAssoPlot (He et al., 2020) was used to visualize the regional GWAS results and the regional LD heatmap.

The physical positions of all genes in each clump interval in GWAS results were extracted from the Williams 82 reference genome. The genotypes of all variants within the interval of each gene of interest, including the 2000-bp upstream regions, were extracted from the soybean population genotype dataset which were generated by variant calling, imputation and annotation pipelines in a previous study, and all cultivars in this population were divided into independent haplotypes based on the different combinations of all variants in the interest region (Zhang et al., 2021). The differences of haplotypes were estimated by ANOVA or Wilcoxon test and visualized by R package ggpubr.

To functionally annotate these interest genes, the encoded protein sequences of these genes were extracted from Williams 82 reference genome, and these sequences were then aligned to *Arabidopsis thaliana* reference genome by BLAST to identified the homologous genes and extract the function of these genes.

## Results

### Phenotypic variation and genetic improvement of 12 agronomic traits

In this study, we planted 496 soybean accessions in two regions (Wuhan and Shijiazhuang) of China during the summer over three consecutive years from 2016 to 2018 (**Supplementary Table 2**). We recorded nine plant architecture traits: plant height (PH), node number (NN), pod range (PR), internode length (IL), lowest pod height (LPH), branch number (BN), the 1^st^ branch angle (1-BA), the 2^nd^ branch angle (2-BA), and the 3^rd^ branch angle (3-BA). We also recorded three seed-related traits: hundred-seed weight (HSW), seed width (SW), and seed length (SL) (**Supplementary Table 3**). The coefficient of variation CV (%) for each of the 12 traits ranged from 5.9% (PR) to 35.4% (PH), highlighting their broad variation among this soybean core collection (**Supplementary Table 4**). Compared to the three seed-related phenotypes, soybean architecture traits were more sensitive to environmental differences (**Supplementary Figure 1**). For example, the CV for 3-BA was greater in Shijiazhuang (28.05%) than in Wuhan (16.80%), while the CV of HSW was 31.03% and 34.74% in Shijiazhuang and Wuhan, respectively (**Supplementary Table 4**). The broad-sense heritability (*H^2^*) of these traits varied from 0.27 (2-BA) to 0.94 (PH and NN). The *H^2^* values of the three seed-related traits were all greater than 0.8, while the three branch angle-related traits exhibited lower heritability values of 0.32, 0.27, and 0.32, respectively (**Supplementary Table 4**). ANOVA indicated that both genotype and environment have significant effects on the 12 traits, and genotype-environment interactions were also highly significant for eight architecture traits with the PR being the sole exception (**Supplementary Table 4**).

**Figure 1:**
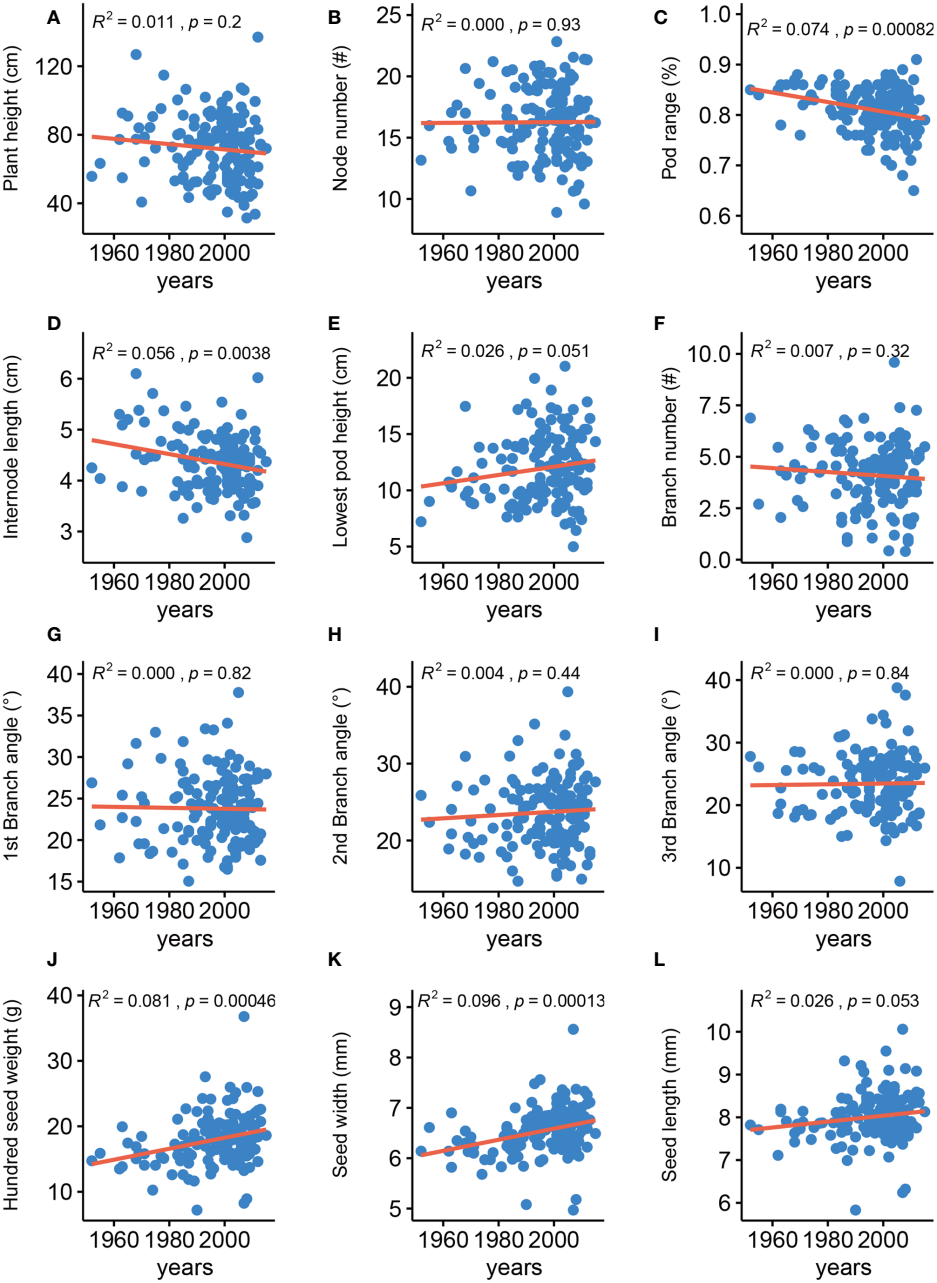
Phenotypic changes of agronomic traits in historical elite soybean varieties. Red line represents linear regression. *R^2^*, *p* calculated by linear regression. **(A)** Plant height, **(B)** Node number, **(C)** Pod range, **(D)** Internode length, **(E)** Lowest pod height, **(F)** Branch number, **(G)** 1st branch angle, **(H)** 2nd branch angle, **(I)** 3rd branch angle, **(J)** Hundred-seed weight, **(K)** Seed width, **(L)** Seed length.

Analyzing the changes in agronomic traits of improved cultivars released in different years of soybean breeding history is important for providing directions for future breeding. We thus analyzed the changes for the 12 traits among 148 soybean varieties that were released from 1952 to 2015 (**Figure 1**), 63% of which are grown in the Huang-Huai-Hai plain region in China (**Supplementary Table 1**). In agreement with an earlier report, we determined that HSW has gradually been increasing (Qin et al., 2017). SW has also gradually been increasing, whereas IL and PR have gradually been decreasing, and the eight remaining traits were generally unchanged. These results indicated that seed-related traits have been the main focus of soybean improvement over the past ~ 60 years in China and that improving architecture traits has the potential for increasing yields in soybean breeding programs.

### Identification of genetic loci controlling 12 agronomic traits via genome-wide association study

**Figure 2:**
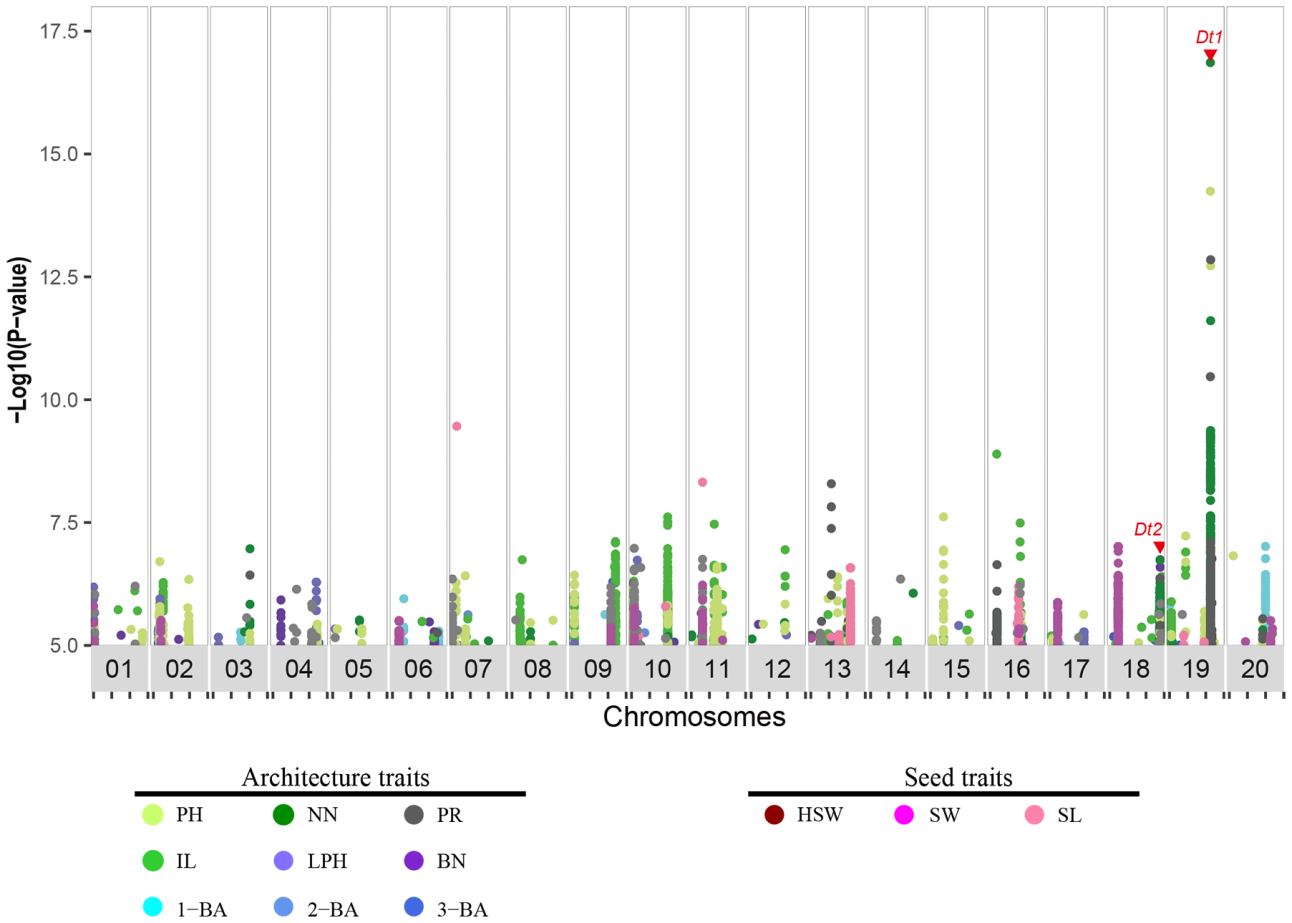
Distribution of genomic association signals for 12 agronomic traits in soybean. Dots in plot indicate markers (SNPs and Indels) detected via genome-wide association study, and significance threshold was set as −log_10_(*p*) = 5. Traits were distinguished by different colors. Red triangle indicates the location of previously characterized genes *Dt1* and *Dt2*. PH, plant height; NN, node number; PR, pod range; IL, internode length; LPH, lowest pod height; BN, branch number; 1-BA, the 1^st^ branch angle; 2-BA, the 2^nd^ branch angle; 3-BA, the 3^rd^ branch angle; HSW, hundred-seed weight; SW, seed width; SL, seed length.

To identify major genetic loci that regulate these 12 traits, we conducted GWAS using the 496-soybean collection. Our analysis was based on the 5,294,054 markers in this natural population and the phenotypes of the 12 traits using seven types of data, comprising phenotypic data from two locations over three years (E1 to E6) and the corresponding best linear unbiased estimator (BLUE) data. Of the GWAS signals, 10,338 (–log_10_ *P* ≥ 5) were significantly associated with the 12 traits (E1:1710, E2:1767, E3:1283, E4:740, E5:1042, E6:1224, BLUE:2572), and 188 signals were repeatedly observed in Wuhan (E1, E3, E5) and the Shijiazhuang (E2, E4, E6) (**Supplementary Table 5**). 786 and 1786 were associated with seed and architecture traits in the BLUE dataset, respectively (**Figure 2** and **Supplementary Figure 2**). To identify the genomic regions defined for all these traits, we used a clumping-based method as previously described (Crowell et al., 2016). We successfully identified 169 genomic regions in the BLUE dataset, of which 129 were associated with architecture traits and 40 were associated with seed-related traits (**Table 1** and **Supplementary Table 6**). Furthermore, we compared all detected genomic regions with previously reported architecture- or yield-related genomic regions or QTLs detected by GWAS or bi-parental QTL mapping, respectively (https://www.soybase.org). We observed that 61 genomic regions co-localize with previously identified QTLs, with the remaining 108 genomic regions being newly identified. Among these genomic regions co-localizing with QTLs, 4 GWAS regions detected for plant height-related traits (PH, NN, IL and PR) overlapped with the previously characterized gene *Dt1*, and the GWAS regions harboring *Dt2* locus were detected for 3 agronomic traits (PH, NN, and BN) (Ping et al., 2014; Fang et al., 2017; Liang et al., 2022). The identification of these overlapping regions (36% of total) indicated that our data collection and analysis were robust. In summary, we identified 108 new genomic regions associated with 12 soybean agronomic traits.

**Table 1:** A subset of QTLs for 12 agronomic traits.

Category	QTL	Traits	-log10 (P)	Chr^a^	Start	End	Environments^b^	Known loci	Linkage QTL^c^	GWAS QTL^d^
Architecture	qBN_1	BN	6.74	18	55019023	56046437	BLUE, E1, E3, E5, E6	*Dt2*		Branching 1-g1.2 Branching 1-g1.1
	qBN_2	BN	5.93	4	8525251	9053777	BLUE, E2			
	qBN_3	BN	5.66	11	10855130	11062033	BLUE, E3, E4			
	qNN_1	NN	16.86	19	44616917	45390253	BLUE, E1, E2, E3, E4, E5, E6	*Dt1*		
	qNN_3	NN	6.73	18	55022904	55785304	BLUE, E1, E3, E4, E6	*Dt2*		Node number 1-g6.1
	qNN_4	NN	5.66	16	33738051	34452508	BLUE, E2, E5			
	qPH_1	PH	14.24	19	44629039	45425935	BLUE, E2, E4, E5, E6	*Dt1*	Plant height 13-8 Plant height 10-4 Plant height 1-1 Plant height 4-2 Plant height 4-4 Plant height 6-1 Stem length, main 2-3 Stem length, main 3-2	Plant height 3-g1 Plant height 5-g3.2 Plant height 3-g2 Plant height 3-g5 Plant height 3-g6 Plant height 3-g9
	qPH_4	PH	6.71	2	6885218	7312480	BLUE, E2, E3, E5, E6		Plant height 23-1 Plant height 13-1	
	qIL_16	IL	5.77	19	44462387	45753882	BLUE, E2, E3, E6	*Dt1*		Interbranch length 1-g1.2 Internode length 1-g2.1 Internode length 1-g2.2 Internode length 1-g2.3
	qPR_1	PR	12.85	19	44967948	45576429	BLUE, E2, E3, E5, E6	*Dt1*		
	qPR_4	PR	6.64	16	7452993	7642598	BLUE, E1, E5, E6			
Seed	qHSW_6	HSW	6.19	9	43031822	43427958	BLUE, E1		Seed weight 27-3	
	qHSW_7	HSW	6.14	4	25700552	26266833	BLUE, E1		Seed weight 36-15 Seed weight 20-2 Seed weight 47-3 Seed weight 45-3	
	qHSW_8	HSW	6.03	1	752381	1281938	BLUE, E1		Seed weight 18-1.2	
	qSL_1	SL	9.46	7	4565298	5119172	BLUE, E1		Seed length 5-3	
	qSL_4	SL	6.09	10	2698397	3037918	BLUE, E1			
	qSL_5	SL	5.79	10	37341548	37690972	BLUE, E1			
	qSW_3	SW	5.88	17	8392962	8940580	BLUE, E1		Seed height 1-8	
	qSW_4	SW	5.80	1	744	451136	BLUE, E1			

a Chr, chromosome; b Environments of QTLs were discovered; c & d QTLs intervals had overlapping as reported in other studies (https://www.soybase.org). E1: Wuhan in 2016, E2: Shijiazhuang in 2016, E3: Wuhan in 2017, E4: Shijiazhuang in 2017, E5: Wuhan in 2018, E6: Shijiazhuang in 2018.

### Construction of a genetic association network for multiple agronomic traits

Correlations between traits is a common phenomenon. Thus, breeders must consider the correlation among traits in variety development, especially when the goal is to increase the values of two negatively correlated traits. We evaluated the phenotypic relationships among the 12 investigated agronomic traits and established that all 12 traits can be grouped into different categories (**Supplementary Figure 3**). Seed traits were strongly and positively correlated (with correlation coefficients ranging from 0.56 to 0.96). For architecture traits, the three branch angle-related traits (1-BA, 2-BA, and 3-BA) were positively correlated (the correlation coefficients ranged from 0.66 to 0.78) and independent from the other traits (with correlation coefficients ranging from 0.01 to 0.08). The four plant height-related traits, PH, NN, IL and PR, were highly and positively correlated, while the same traits were negatively correlated with HSW and SW (with correlation coefficients ranging from -0.29 to -0.50) (**Supplementary Figure 3**). These results suggested that regulating plant height-related traits might have negative effects on seed width and seed weight. By contrast, optimizing branch angle-related traits might have minor effects on seed-related traits in soybean.

**Figure 3:**
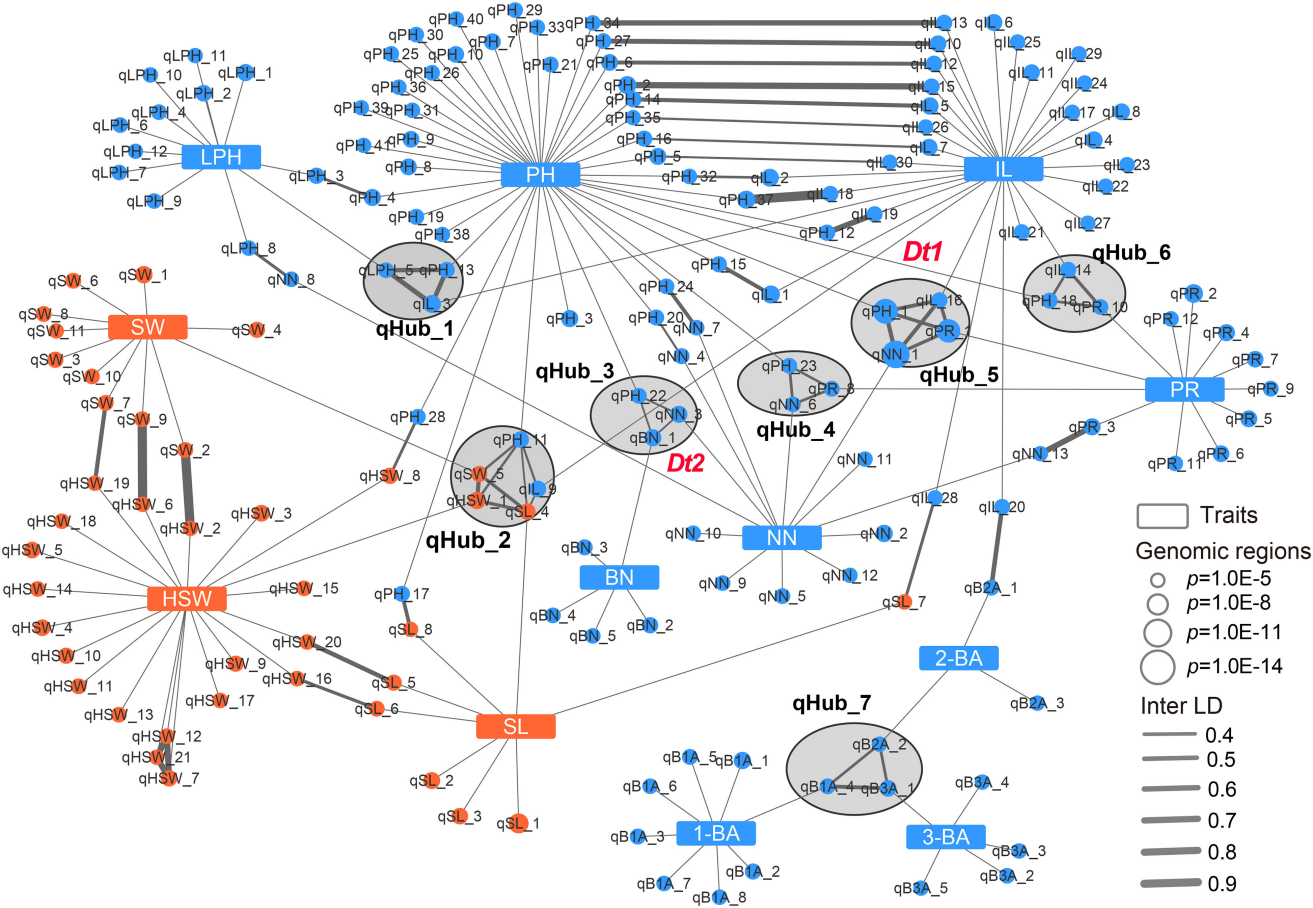
Genetic association network of 12 agronomic traits in soybean. Blue rectangles and blue circular nodes represent architecture traits and architecture-related genomic regions, respectively. Orange rectangles and orange circular nodes represent seed traits and seed-related genomic regions, respectively. Lines show the connection between traits and genomic regions or linkage disequilibrium of genomic regions (inter-LD). Black circles filled gray indicate the hub-nodes which correlate to multiple traits. PH, plant height; NN, node number; PR, pod range; IL, internode length; LPH, lowest pod height; BN, branch number; 1-BA, the 1^st^ branch angle; 2-BA, the 2^nd^ branch angle; 3-BA, the 3^rd^ branch angle; HSW, hundred-seed weight; SW, seed width; SL, seed length.

The above results indicated that phenotypic correlations were common for soybean agronomic traits. In particular, *Dt1* was associated not only with PH and NN, but also with IL and PR, and the *Dt2* locus was simultaneously detected for PH, NN, and BN, suggesting that these related traits might be genetically co-regulated. Association networks allow regions of the genome associated with multiple phenotypes to be quantitatively identified. Therefore, we built a genetic association network by integrating the above GWAS results to explore the genetic relationship of the target traits. In total, we included 169 genomic regions defined by GWAS in this network, 90 of which were associated with a single agronomic trait, while the remaining 79 were linked to other regions by Inter-linkage disequilibrium (LD) or different traits. Of these 79 regions, 26 were associated with two traits, and 7 hub-nodes that linked to at least three traits were present in the multiple trait association network (**Figure 3**). Notably, 12 and 6 GWAS regions were solely associated with IL and NN, respectively, suggesting that several relatively independent loci involved in regulating plant height-related traits were identified in this study (**Figure 3**). qHub_2 is a newly identified hub-node linking three seed-related traits (HSW, SW and SL) and two plant height-related traits (PH and IL) (**Figure 3** and **Supplementary Table 7**). This genetic association might partially explain the significant phenotypic correlation between seed-related traits and plant height-related traits.

*Dt1* and *Dt2* are two key genes regulating plant height and node number in soybean, and they were presented in two hub-nodes, qHub_5 and qHub_3, respectively (**Figure 3**). In agreement with previous reports, in this network, the *Dt2* locus overlapped with a pleiotropic locus (qHub_3) associated with PH, NN, and BN (Ping et al., 2014; Fang et al., 2017; Liang et al., 2022). *Dt1* was reported to affect plant height, node number, branch density, stem pod density, the number of three-seeded pods, and total seed number (Fang et al., 2017). In the association network, qHub_5 (containing *Dt1*) was linked to four plant height-related traits: PH, NN, IL and PR (**Figure 3**). To investigate whether *Dt1* might affect IL and PR, we conducted haplotype analysis for above plant height-related traits based on previously reported *Dt1* haplotypes information (H1, H2, H3, H4) that were detected in 496 soybean accessions (Liu et al., 2010; Tian et al., 2010; Yue et al., 2021). Cultivars with the *Dt1*-H1 haplotype carrying the functional *Dt1* allele were taller and produced more nodes than those carrying the *dt1* haplotypes (H2, H3 and H4), as reported previously (Yue et al., 2021). Moreover, cultivars with the functional *Dt1* allele had longer internodes and wider pod ranges than those with the *dt1* alleles (H2, H3) (**Supplementary Figure 4**). In addition, according to the released transcriptome data retrieved from the Plant Public RNA-seq Database (https://plantrnadb.com/), *Dt1* is expressed in axillary meristems and internodes (**Supplementary Figure 5**), suggesting that *Dt1* might also affect soybean internode length in addition to node number and plant height.

### Analysis of candidate genes in qHub_7 for branch angle traits

**Figure 4:**
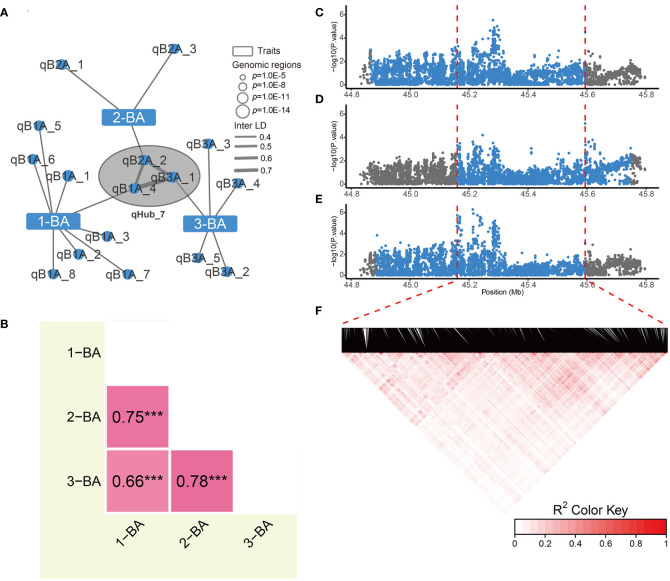
Phenotypic correlations and hub node details for genetic association networks of branch angles. **(A)** The association networks across 1-BA, 2-BA and 3-BA. The Hub-node of branch angle traits indicated by the black circle filled with gray. **(B)** Phenotypic correlations of 3 branch angle traits. The numbers indicate pearson’s correlation coefficient between two traits, and *** indicates *p*-value< 0.001. **(C-E)** Regional manhattan plots of the 1^st^ **(C)**, 2^nd^ **(D)** and 3^rd^ **(E)** branch angle GWAS results, respectively. Blue points indicate the variations within candidate region for each trait. Red dashed lines indicate the border of the overlapped genomic region. **(F)** Linkage disequilibrium heatmap of variations in overlapped region for 3 branch angle traits.

Optimizing genes that regulate tiller/leaf angles has greatly contributed to boosting maize and rice yields under high-density planting (Li et al., 2007; Tian et al., 2019; Huang et al., 2021). To date, only a few studies have focused on genetically modifying soybean branch angle traits (Clark et al., 2021). Moreover, correlation analysis showed that branch angle traits were independent from other traits (**Supplementary Figure 3**). In the genetic association network, three branch angle traits are associated with 16 genomic regions, and only one genomic region associated with 2-BA was linked to internode length by Inter-LD (value of Inter-LD is 0.6) (**Figure 3** and **Figure 4A**). We also discovered a hub-node region (qHub_7) for branch angle traits. Consistent with our correlation analysis (**Supplementary Figure 3**), the genetic association network indicated that branch angle traits were relatively independent and were strongly and positively correlated to each other as compared with the nine other agronomic traits (**Figure 3**).

Since these three branching phenotypes were similar and closely and positively correlated (**Figure 4B**), we reasoned that candidate genes regulating these three traits are most likely present in qHub_7. The interval lengths of the GWAS regions for 1-BA, 2-BA, and 3-BA were 728 kb, 588 kb, and 706 kb, respectively (**Figures 4C–F**). We extracted the sequences of all genes in each region and performed haplotype analysis for the corresponding branch angles. We identified 16, 26, and 19 genes that were significantly associated with phenotypic differences among the haplotypes of these genes for 1-BA, 2-BA, and 3-BA, respectively. We considered the overlapping genes among these gene sets (eight genes) to be interesting candidates for branch angle-related traits (**Supplementary Table 8** and **Supplementary Figure 6**). We then searched a public gene expression database for the expression patterns of these candidate genes in different tissues and developmental stages (Shen et al., 2014). Only four genes (*Glyma.09G227100*, *Glyma.09G228200*, *Glyma.09G228300*, and *Glyma.09G229000*) were expressed, whereas the four other genes, whose expression was not detected, were excluded from further analysis (**Supplementary Figure 7**).

To narrow down the list of candidate genes regulating branch angle, we examined data from functional studies of their homologs in *Arabidopsis thaliana*. The homologs of *Glyma.09G228300* in Arabidopsis have no known functions and are annotated as encoding a protein with unknown function (DUF3411) and an uncharacterized protein. *Glyma.09G227100* encodes a UvrABC system C protein involved in DNA damage repair. *Glyma.09G229000* encodes a homolog of Arabidopsis actin-related protein 7 (ARP7) according to TAIR (https://www.arabidopsis.org/). *ARP7* plays vital roles in embryogenesis and plant development, as knocking down this gene in Arabidopsis led to dwarfism, small rosette leaves, retarded root growth, altered flower development, delayed perianth abscission, and reduced fertility (Kandasamy et al., 2005). Intriguingly, *Glyma.09G228200* encodes a protein homologous to the FK506-binding protein 42 (FKBP42) protein TWISTED DWARF 1 (ATWD1). *TWD1* is involved in the auxin signal transduction pathway as well as brassinosteroid (BR) signaling (Wu et al., 2010; Zhao et al., 2016; Zhu et al., 2016). Both auxin and BRs play vital roles in modulating tiller or leaf angle in rice or maize (Sun et al., 2015; Tian et al., 2019; Wang et al., 2020b). The Arabidopsis *twd1* mutant shows drastically reduced cell elongation combined with a disoriented growth behavior (Geisler et al., 2003). Thus, these results suggested that *Glyma.09G228200* and *Glyma.09G229000* are most likely involved in branch angle regulation. It will be necessary to genetically manipulate these two genes in soybean in order to verify their functions.

## Discussion

Introducing the genes for multiple desirable traits in a single genome is an ideal goal of crop breeding. However, agronomic trait correlations are commonly observed in breeding programs. Gene pleiotropy and LD are major reasons for genetic trait correlations (Chen and Lubberstedt, 2010). In this study, the genetic association network generated provides global insights into the genetic relationships of 12 agronomic traits of soybean. Ninety genomic regions that were linked to single traits provided potential opportunities for breeders to break undesirable phenotypic correlations and modulate single traits independently with only slightly negative effects. In addition, we identified 7 hub-nodes linked to multiple related traits in the association network. Further studying the functional genes of these hub-nodes will shed light on the nature of genetic correlations for these traits.

Soybean plants with ideal architecture required more nodes and shorter internodes than typical plants. In this study, we found that in addition to node number (Liu et al., 2010; Tian et al., 2010), *Dt1* was also associated with internode length (**Figure 3** and **Supplementary Figure 4**). However, correlation analysis showed that node number was highly and positively correlated with internode length. Due to this strong correlation, it is not easy to break the complex trade-offs caused by *Dt1* pleiotropy. These findings suggest that if breeders hope to utilize the *Dt1* locus to increase node number in soybean, the risk of lodging must be taken into consideration. In fact, in addition to the three classical types of soybean stem termination architectures (indeterminate, determinate, and semi-determinate), Thompson identified an additional stem termination type termed tall determinate (delayed cessation of the apical stem after flowering), and the gene controlling the tall determinate phenotype is *dt1-t*, which is allelic to *dt1* (Thompson et al., 1997). In a recent study, compared to typical determinate soybeans carrying the *dt1* allele (R166W), tall determinate soybeans carrying *dt1-t1* (R130K) or *dt1-t2* (R62S) showed potential agronomic merits, including an increased number of pod-bearing nodes and enhanced lodging resistance (with a similar stem diameter) (Kim et al., 2022). The previous and current results in this study suggest that different alleles of *Dt1* might have different effects on plant height-related traits (**Supplementary Figures 4B, C**). Despite the importance of *Dt1* in regulating soybean traits, six nonsynonymous variants have been identified in the coding region of *Dt1* (R62S, P113L, L67Q, P113L, R130K, H141R) (Liu et al., 2010; Tian et al., 2010; Liu et al., 2015; Yue et al., 2021). However, the specific effects of each *Dt1* allele and the possible differences in soybean architecture conferred by these mutations have not yet been defined. Therefore, it would be worthwhile to further study the effects of *Dt1* allelic variants on the agronomic traits of soybean, as these variants have the potential to improve yields by generating plants with more pod-bearing nodes along with lodging resistance.

Compact plant architecture (tiller/branch angle and leaf erectness) facilitates dense planting to boost yields. Upright architecture traits have been studied in rapeseed (*Brassica napus*), cotton (*Gossypium hirsutum*), and cereal crops (Sun et al., 2015; Cheng et al., 2017; Tian et al., 2019; Wang et al., 2020a; Wang et al., 2020b; Ji et al., 2021). However, few reports on branch erectness in soybean are currently available (Clark et al., 2021). In this study, we investigated the 1^st^, 2^nd^, and 3^rd^ branch angle traits in a natural population of soybeans grown in two environments and determined that all three branch angle traits exhibited a large range of natural variation. However, in contrast to other agronomic traits, we detected only 13 genomic regions by GWAS for three branch angle traits. None of these regions were repeatedly observed in the environmental dataset (Wuhan and Shijiazhuang) or BLUE data, and the broad-sense heritability (*H^2^*) for branch angle traits was only approximately 0.30. It is possible that branch angle traits are significantly affected by many other environmental factors, such as planting density, sowing date, or other agronomic practices, making it difficult to study branch angle traits using GWAS (Clark et al., 2021). We also observed that branch angle traits were relatively independent and were strongly correlated to each other. The association network contains the hub-node qHub_7, which controls three branch angle-related traits; this hub-node may serve as a target for breeding soybean with compact architecture.

In summary, we explored the genetic basis of plant architecture and seed traits in soybean. We constructed a multiple trait association network and predicted seven hub-nodes associated with ideal soybean architecture. This information will facilitate the genetic improvement for higher yield via soybean breeding programs.

## Data availability statement

The original contributions presented in the study are included in the article/supplementary material. Further inquiries can be directed to the corresponding authors.

## Author contributions

MN: Data curation, Formal analysis, Investigation, Methodology, Software, Validation, Visualization, Writing – original draft, Writing – review & editing. KT: Data curation, Formal analysis, Investigation, Methodology, Software, Validation, Visualization, Writing – original draft, Writing – review & editing. QC: Writing – review & editing, Data curation. CY: Data curation, Writing – review & editing. MZ: Data curation, Formal analysis, Writing – review & editing. SS: Funding acquisition, Project administration, Resources, Supervision, Writing – original draft, Writing – review & editing. XW: Funding acquisition, Project administration, Resources, Supervision, Writing – original draft, Writing – review & editing.
