# Supplementary material for: A multi-trait GWAS-based genetic association network controlling soybean architecture and seed traits
Source: Front Plant Sci. 2024 Jan 8;14:1302359. doi: 10.3389/fpls.2023.1302359 (PMC10801003; doi:10.3389/fpls.2023.1302359)
Supplement: Supplementary file 1 [file DataSheet_1.pdf]

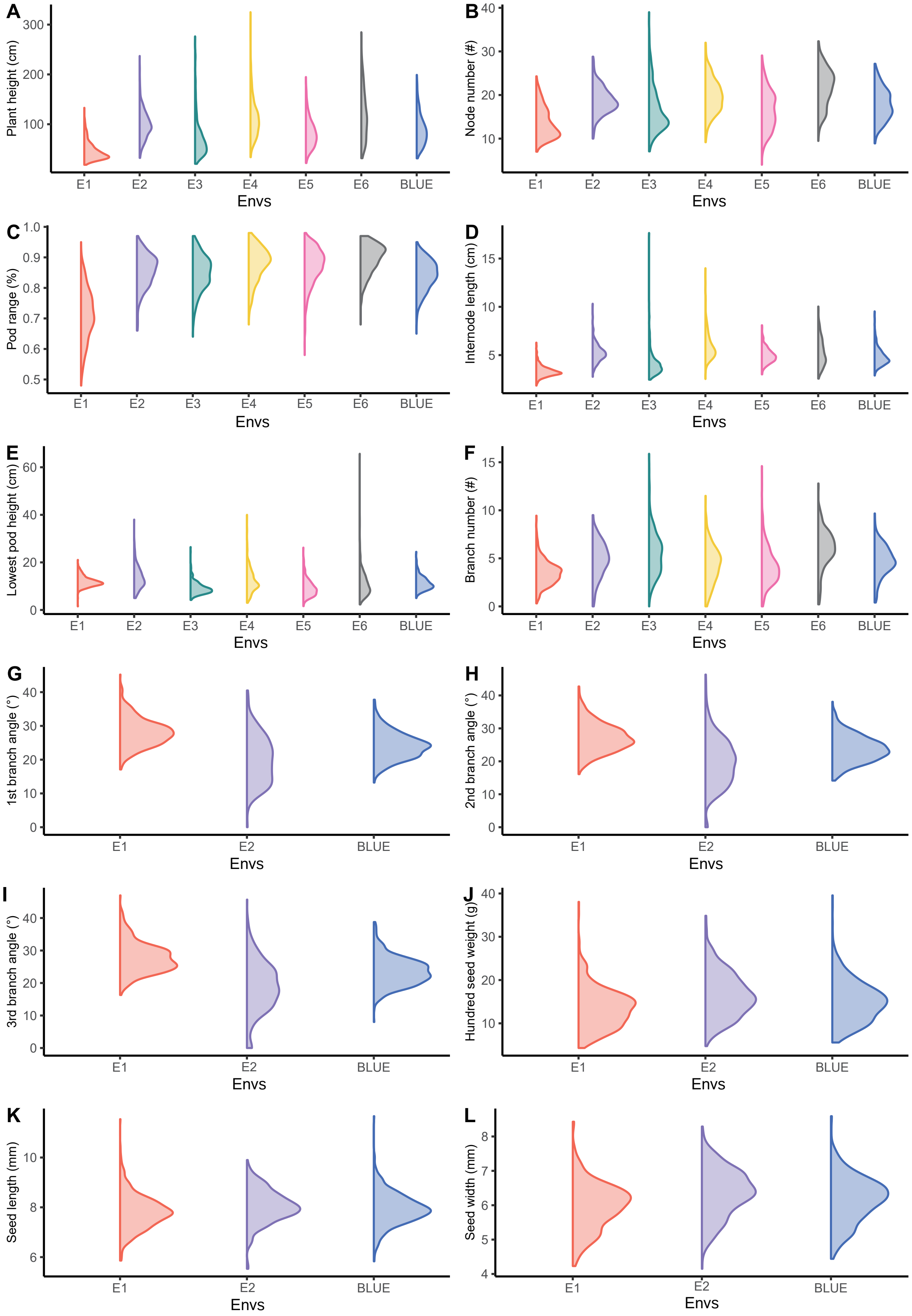

FIGURE S1 | The distribution of phenotypes for 12 traits in multiple environments.

Envs indicates different environments for combination of locations and years. E1: WH_2016; E2: SJZ_2016; E3: WH_2017; E4: SJZ_ 2017;

E5: WH_2018; E6: SJZ_2018.

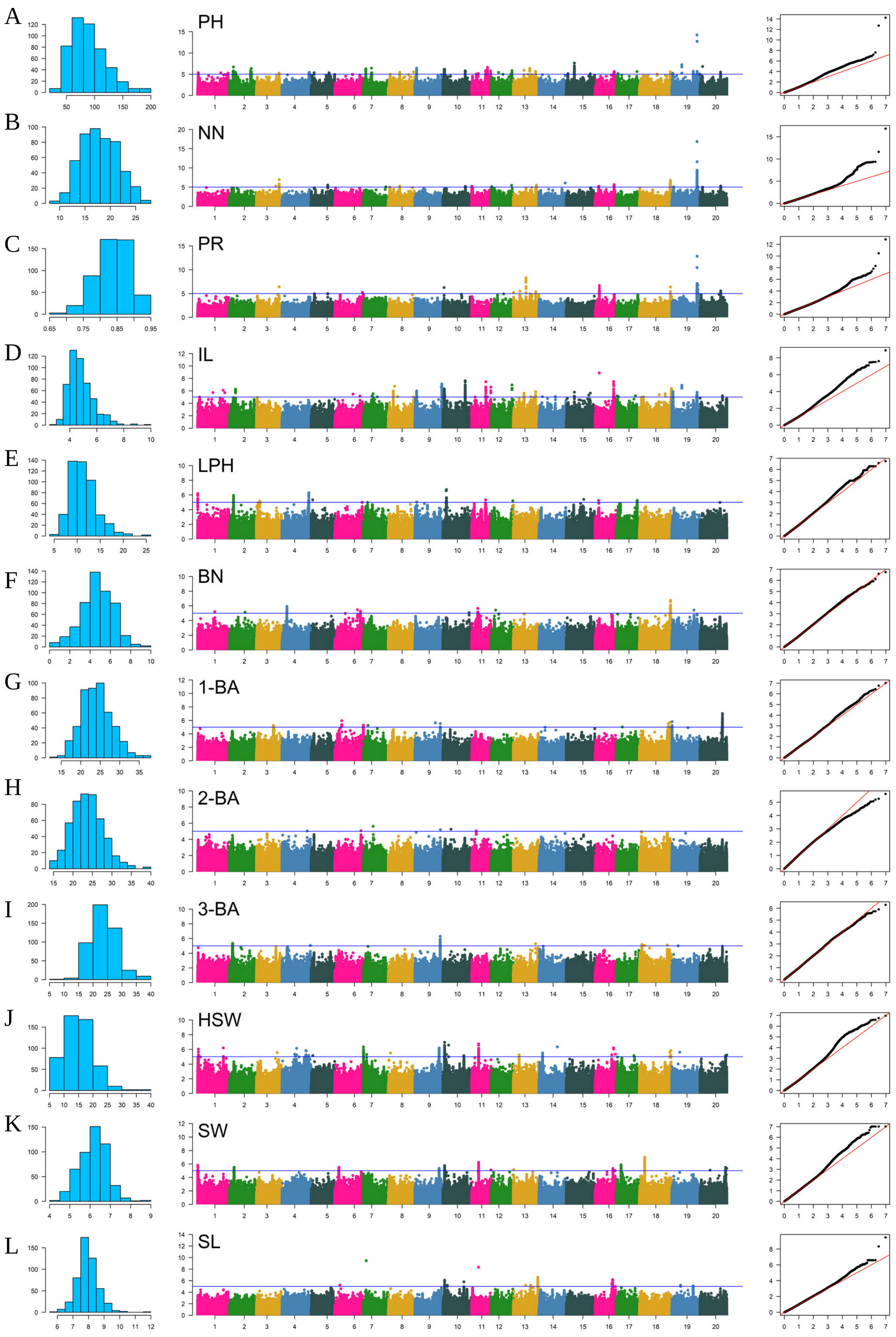

FIGURE S2 | GWAS results of 12 agronomic traits.
Left panel displayed the frequency distribution of each trait. Central panel displayed the GWAS results of each trait with manhattan plot. Right panel displayed the QQ plot of each trait. All results calculated based on BLUE data.

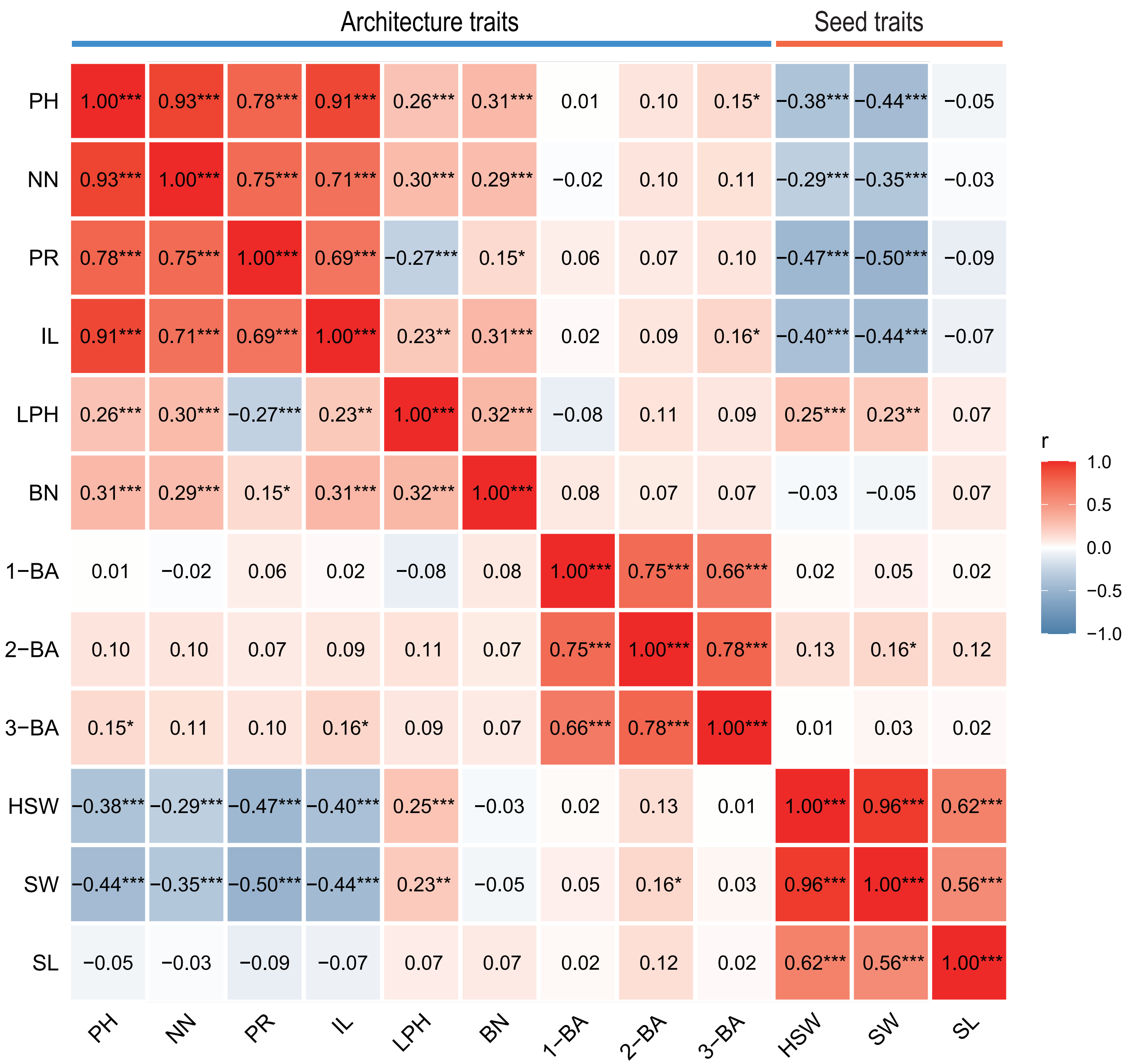

FIGURE S3 | Correlation analysis of 12 agronomic traits.
The numbers indicate pearson's correlation coefficient between two traits, and *p*: *** 0.001, ** 0.01, * 0.05. The 12 traits were classified into two sub-groups: Architecture and Seed traits.

A

Reference	Alleles in previous report	*Dt1* haplotypes in this study	Site 1	Site 2	Site 3	Counts
W82	*Dt1*	H1	G	C	A	307
	dt1-ta	H2	T	C	A	73
	dt1-bb	H3	G	C	T	56
	dt1-ab	H4	G	T	A	54
CDS nt # in W82			186	338	496	
AA # in W82			62	113	166	
AA identified in W82			R	P	R	
AA change			S	L	W	

B

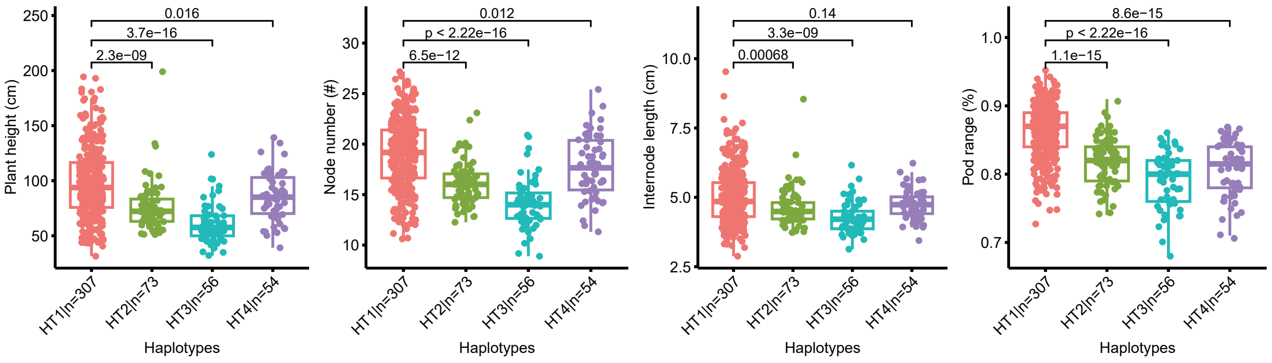

C

Plant height (cm)	HT2	HT3	HT4
HT1|n=307	2.3e−09	3.7e−16	0.0160
HT2|n=73		1.2e−06	0.0027
HT3|n=56			9.6e−09

Node number (#)	HT2	HT3	HT4
HT1|n=307	6.5e−12	< 2e−16	0.0124
HT2|n=73		3.0e−07	0.0022
HT3|n=56			4.2e−09

Internode length (cm)	HT2	HT3	HT4
HT1|n=307	0.00068	3.3e−09	0.13536
HT2|n=73		0.00027	0.06705
HT3|n=56			5.7e−06

Pod range (%)	HT2	HT3	HT4
HT1|n=307	1.1e−15	< 2e−16	8.6e−15
HT2|n=73		0.0002	0.2697
HT3|n=56			0.0355

FIGURE S4 | Phenotypic comparison of *Dt1* haplotypes in different phenotypic datasets.
(A) Nucleotide polymorphisms in the coding region of *Dt1* in our core population. (B) The phenotypic differences of 3 *dt1* alleles compared to *Dt1* allele in PH, NN, IL and PR. The significance were calculated by wilcoxon test. (C) The tables of phenotypic comparison between each pair of haplotypes in PH, NN, IL and PR. The diagonal number indicate the mean value of each haplotype in these phenotypic dataset. The significance were calculated by wilcoxon test.

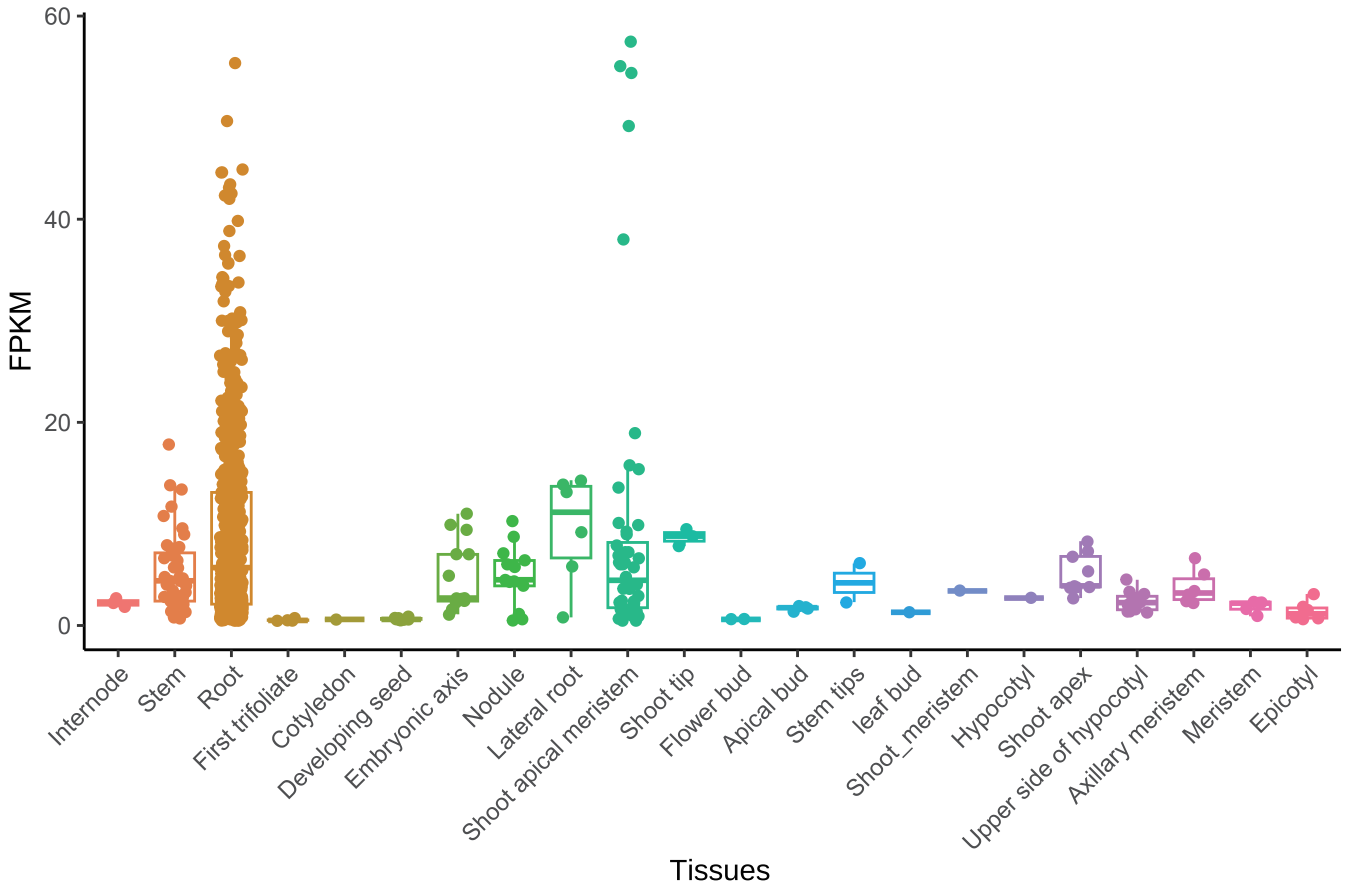

FIGURE S5 | *Dt1* expression level in multiple tissues.
Dt1 expression data retrieved from the Plant Public RNA-seq Database (https://plantrnadb.com/), which shows the expression of *Dt1* can be detected in axillary meristem and internode.

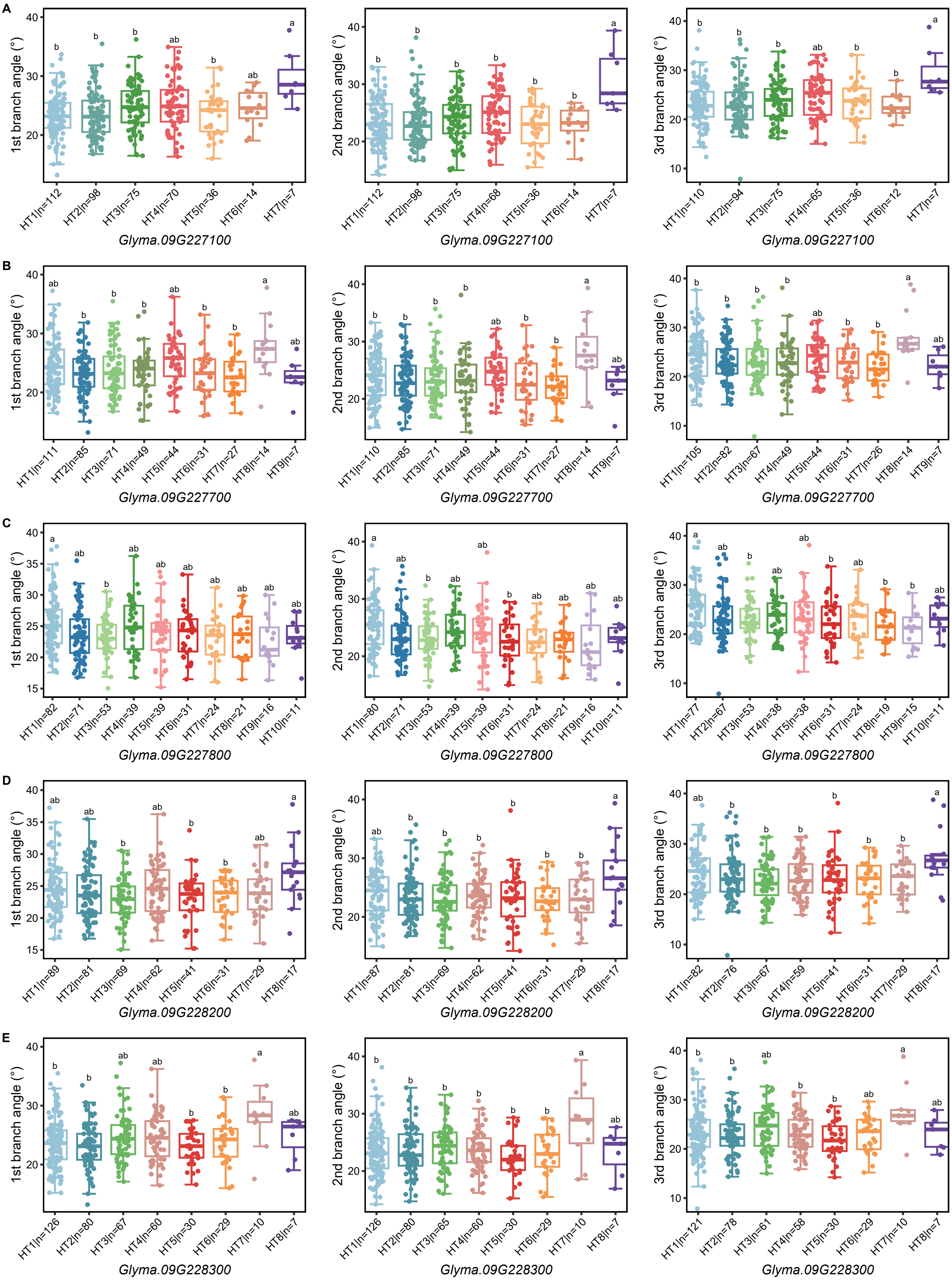

A
B
C
D
E
1st branch angle (°)
2nd branch angle (°)
3rd branch angle (°)
HT1|n=112 HT2|n=98 HT3|n=75 HT4|n=70 HT5|n=36 HT6|n=14 HT7|n=7
HT1|n=112 HT2|n=98 HT3|n=75 HT4|n=68 HT5|n=36 HT6|n=14 HT7|n=7
HT1|n=110 HT2|n=94 HT3|n=75 HT4|n=65 HT5|n=36 HT6|n=12 HT7|n=7
Glyma.09G227100
HT1|n=111 HT2|n=85 HT3|n=71 HT4|n=49 HT5|n=44 HT6|n=31 HT7|n=27 HT8|n=14 HT9|n=7
HT1|n=110 HT2|n=85 HT3|n=71 HT4|n=49 HT5|n=44 HT6|n=31 HT7|n=27 HT8|n=14 HT9|n=7
HT1|n=105 HT2|n=82 HT3|n=67 HT4|n=49 HT5|n=44 HT6|n=31 HT7|n=26 HT8|n=14 HT9|n=7
Glyma.09G227700
HT1|n=82 HT2|n=71 HT3|n=53 HT4|n=39 HT5|n=39 HT6|n=31 HT7|n=24 HT8|n=21 HT9|n=16 HT10|n=11
HT1|n=80 HT2|n=71 HT3|n=53 HT4|n=39 HT5|n=39 HT6|n=31 HT7|n=24 HT8|n=21 HT9|n=16 HT10|n=11
HT1|n=77 HT2|n=67 HT3|n=53 HT4|n=38 HT5|n=38 HT6|n=31 HT7|n=24 HT8|n=19 HT9|n=15 HT10|n=11
Glyma.09G227800
HT1|n=89 HT2|n=81 HT3|n=69 HT4|n=62 HT5|n=41 HT6|n=31 HT7|n=29 HT8|n=17
HT1|n=87 HT2|n=81 HT3|n=69 HT4|n=62 HT5|n=41 HT6|n=31 HT7|n=29 HT8|n=17
HT1|n=82 HT2|n=76 HT3|n=67 HT4|n=59 HT5|n=41 HT6|n=31 HT7|n=29 HT8|n=17
Glyma.09G228200
HT1|n=126 HT2|n=80 HT3|n=67 HT4|n=60 HT5|n=30 HT6|n=29 HT7|n=10 HT8|n=7
HT1|n=126 HT2|n=80 HT3|n=65 HT4|n=60 HT5|n=30 HT6|n=29 HT7|n=10 HT8|n=7
HT1|n=121 HT2|n=78 HT3|n=61 HT4|n=58 HT5|n=30 HT6|n=29 HT7|n=10 HT8|n=7
Glyma.09G228300

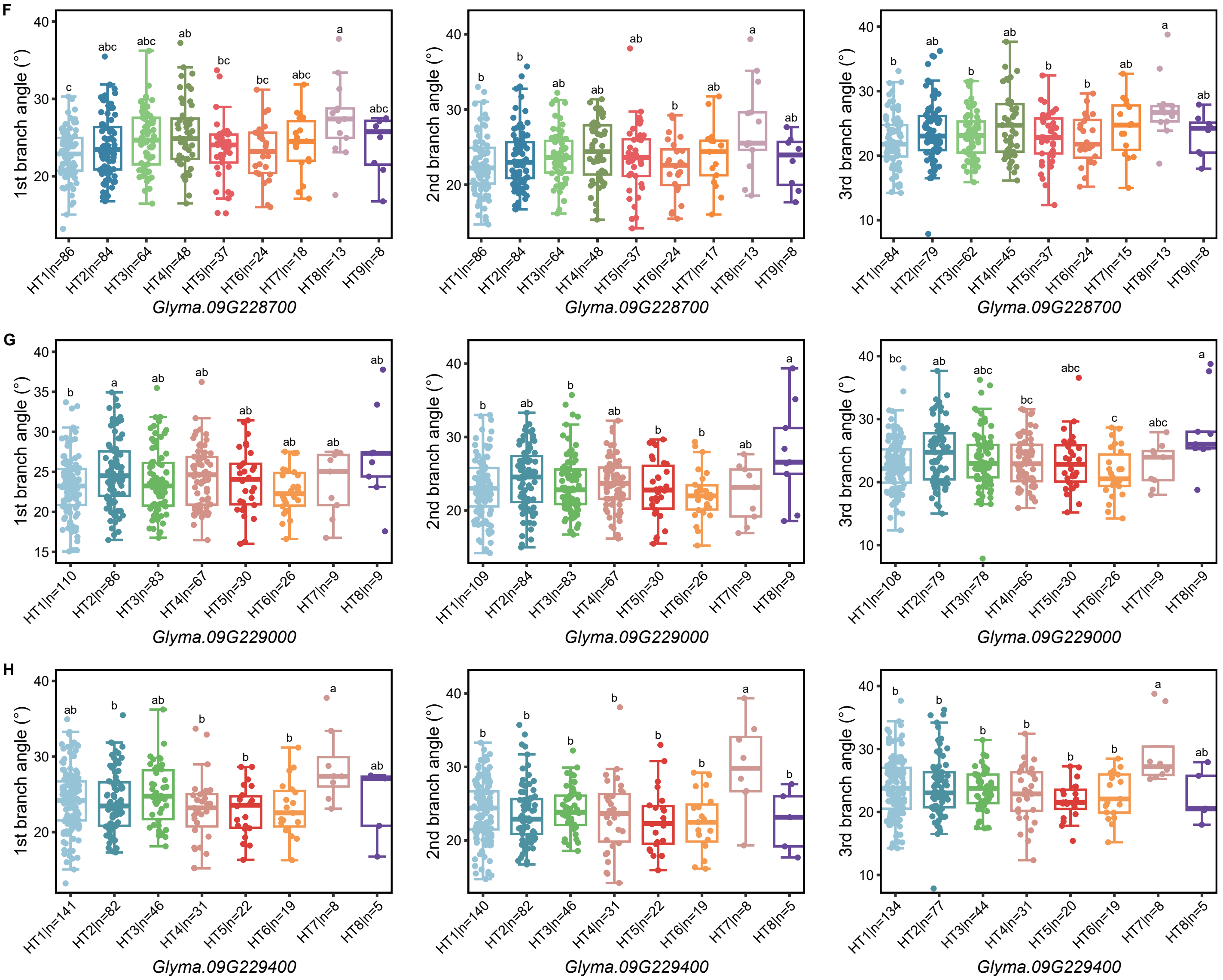

FIGURE S6 | Haplotypes and phenotypic analysis of branch angle traits for 8 candidate genes in qHub_7. These genes showed significant phenotypic difference between some haplotypes in all three branch angle traits. The phenotypic differences were determined by ANOVA.

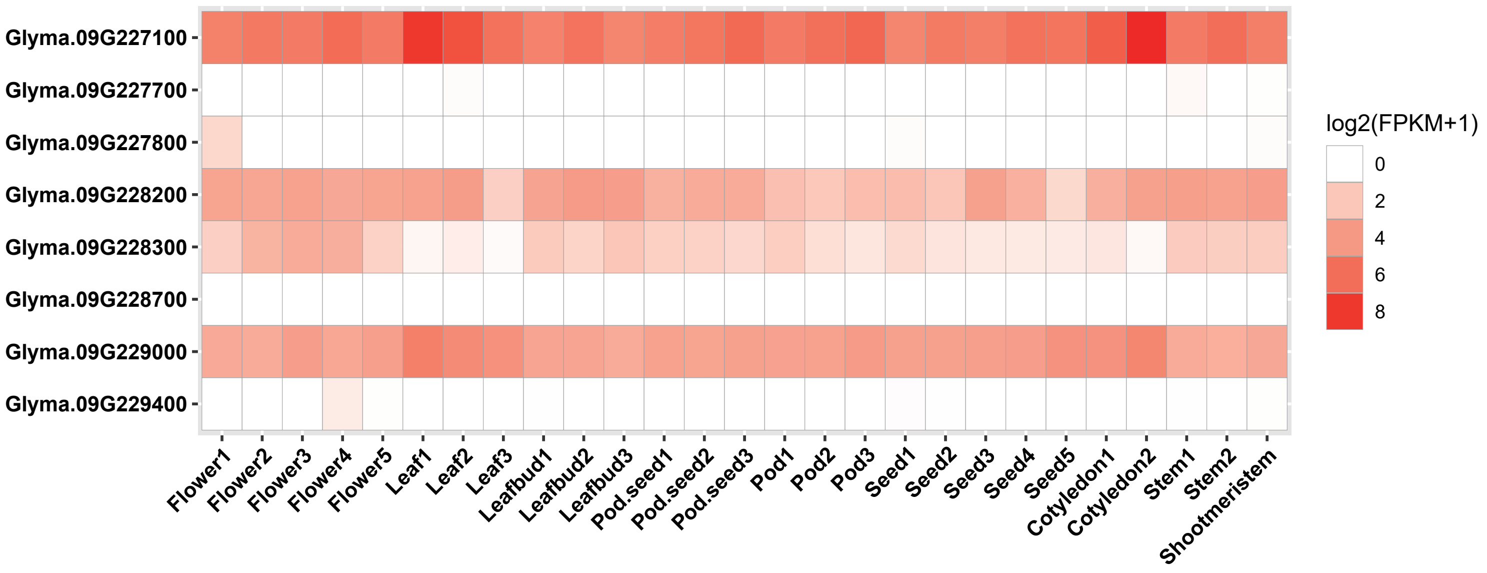

FIGURE S7 | Expression pattern of all 8 candidate genes in qHub_7. The expression data was retrieved from previous published transcriptome data (Shen et al., 2014)
